# Dodging the bullet: therapeutic resistance mechanisms in pediatric cancers

**DOI:** 10.20517/cdr.2019.20

**Published:** 2019-09-19

**Authors:** Nilay Shah

**Affiliations:** ^1^Division of Pediatric Hematology/Oncology/BMT, Nationwide Children’s Hospital, Columbus, OH 43205, USA.; ^2^Department of Pediatrics, The Ohio State University College of Medicine, Columbus, OH 43210, USA.

**Keywords:** Tumor microenvironment, drug efflux, chemoresistance, inhibition of apoptosis, BCL2, pediatric cancer

## Abstract

While advances in the treatment of pediatric cancers have improved survival to > 80% across all tumor types, drug resistance continues to limit survival for a considerable number of patients. We review the known mechanisms of resistance in pediatric cancers, including processes that impair conventional chemotherapies, newer classes of targeted small molecule antineoplastic drugs, and monoclonal antibodies. We highlight similarities and differences in treatment approach and resistance between pediatric and adult cancers. We also discuss newer areas of research into drug resistance, including extracellular and immune factors.

## Introduction

Pediatric cancer therapies have made significant advances over the last 70 years, with 5-year overall survival rates rising from < 20% in the 1960’s to > 80% today^[1]^. Those improvements have not been uniform, however, across pediatric cancer histologies, including significant mortality among patients with acute myeloid leukemia, high-risk neuroblastoma, metastatic sarcomas, and specific brain tumors^[1,2]^. Resistance to cancer therapies, including chemotherapies and radiation therapy, have been an area of study for many decades, identifying some key mechanisms that allow cancer cells to remain viable. The development of new therapeutic approaches, including tyrosine kinase inhibitors, monoclonal antibodies, and immunoncology approaches, have resulted in yet more resistance mechanisms. These various mechanisms include molecular changes at the intracellular, transcellular, and intercellular levels, and include effects at the genetic, epigenetic, transcriptional and translational levels.

Here, we review the various mechanisms of therapeutic resistance against cancer therapies. We begin with a review of common mechanisms of action of traditional antineoplastic therapies, including key pathways that can be exploited by cancer cells. We will then review established mechanisms of resistance to classical treatments, exploratory studies into resistance against modern targeted agents, and new directions of research into cancer cell viability, as they apply to childhood cancers. We specifically highlight similarities and differences in therapy resistance in pediatric and adult cancers, as well as some important approaches being taken by pediatric oncologists and researchers to tackling treatment resistance.

## The battlefield of the cancer cell

Traditional chemotherapy and radiation therapy act through diverse mechanisms, including inhibition of cell division kinetics, inhibition of DNA replication, direct DNA damage, and metabolic inhibition. However, there are some cellular activities that must exist for these agents to act against cancer cells. First, the drug must be stably delivered to the cell through the blood supply^[3]^; some drugs additionally need to be converted from the prodrug to the active agent either at the liver^[4,5]^ or, less commonly, in the target cell^[6]^. Second, the drug must be able to enter the cell, either through passive diffusion^[7,8]^ or more commonly through exploitation of existing transport mechanisms^[9]^. Third, the drug must be retained in the cells and reach its target. Fourth, the drug must be able to bind its target. Fifth, the activity of the agent must be able to induce cytotoxicity; this can occur through a number of mechanisms, including intrinsic induction of apoptosis through DNA damage signaling and/or cytochrome C-mediated apoptosis, or through extrinsic activation of caspase-mediated apoptosis. Optionally, the agent must be able to pause cell proliferation by inhibiting cell cycling.

As an example, we can review doxorubicin-mediated cytotoxicity. Doxorubicin is an anthracycline antibiotic that can be derived from Streptomyces species, though it is most commonly generated through recombinant methods today. It is used against a number of childhood cancers including leukemias, lymphomas, and solid tumors^[9]^. Doxorubicin is a vesicant, so it is administered through central venous catheters directly into the bloodstream, where it is delivered to its targets. It can passively enter the cell but more commonly via the transmembrane channel SLC22A16^[10]^. Once in the cell, it can activate apoptosis either by DNA intercalation, activating DNA damage pathways, or by generation of reactive oxygen species, causing membrane damage and cytochrome C-mediated apoptosis^[11]^. Doxorubicin also induces G2 arrest through interference with DNA replication^[12]^. The activity of doxorubicin identifies key nodes of toxicity that cancer cells can then modify to maintain viability.

## Specific considerations of pediatric cancers and pediatric cancer therapy

Just as children are not simply small adults, pediatric cancers and their treatment have significant differences from adult tumors. Whereas adult cancers, particularly carcinomas, harbor high rates of gene mutations and fusion oncogenes, pediatric cancers are comparatively genomically “quiet”^[13]^. Epigenetic dysregulation, due to various mechanisms including mutations or aberrant imprinting, seems to be of particular import in many types of pediatric cancers (reviewed in^[14-20]^). Additionally, segmental chromosomal changes, including segmental gains, losses, and translocations, are observed in multiple pediatric cancers and correlate with disease severity^[21-30]^, suggesting that the key genetic changes that lead to malignancy may occur in a more cataclysmic fashion than in adult cancers. These features have had therapeutic implications historically and with regard to new drug development.

**Figure 1:**
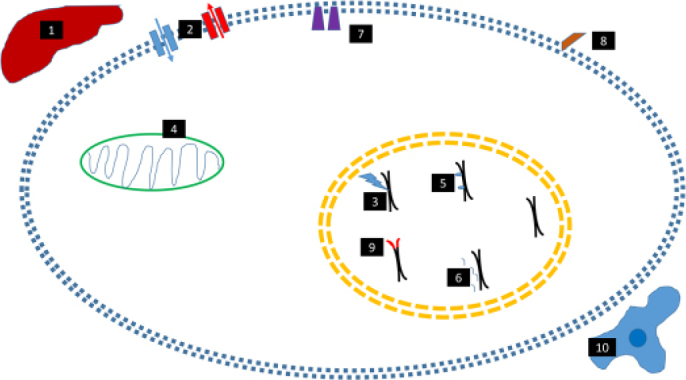
Mechanisms of therapeutic resistance in pediatric cancers. (1) Altered drug metabolism, either at the liver, in the bloodstream, or in the cell; (2) Altered drug influx/effux; (3) Disruption of response to DNA damage; (4) Inhibition of mitochondrial/cytochrome c/ROS-mediated cell death; (5) Epigenetic dysregulation; (6) miRNA dysregulation; (7) Mutations/amplification of tyrosine kinases; (8) Abnormal expression/downregulation of cell surface markers and targets of monoclonal antibodies and other immune therapies; (9) Activation of telomerase/ALT. (10) Tumor microenvironment enabling viability

Pediatric cancer treatment regimens, like most cancers, were developed since the 1940’s^[31]^ and based on the use of conventional chemotherapies that targeted key processes of DNA replication and cell division. Since then, two hallmarks of treatment have been (1) systemic chemotherapy use, with some augmentation with external beam radiation therapy and/or surgery; and (2) the use of multiagent regimens with dose intensification. The former was based on evidence of hematologic or lymphatic disease dissemination, as exemplified in patients with localized osteosarcoma who had metastatic recurrence despite surgery alone^[32]^. The latter led to marked improvements in long-term cures across pediatric tumor types (reviewed here^[2]^), in part by overcoming some of the resistance mechanisms described below. These approaches were possible due to increased hematopoietic recovery in children as compared to adults, improvements in supportive care including blood product transfusions, infection control measures, better baseline organ function in children, and a tolerance of heightened toxicity as a cost of improved survival^[33]^. This has resulted in long-term morbidity for survivors, including effects on growth, neurocognitive development, endocrine health, predisposition to metabolic syndrome, cardiotoxicity, pulmonary toxicity, nephrotoxicity, fertility issues, and secondary malignancy risk; these have been reviewed more comprehensively elsewhere^[34-40]^. These outcomes emphasize how the identification of treatment resistance mechanisms and resultant counterstrategies are needed to reduce treatment intensity and toxicity while improving survival, which we now discuss [Figure 1].

## Cellular mechanisms of therapeutic resistance and counterstrategies

### Drug metabolism

A significant number of chemotherapeutic drugs used in pediatric cancers are actually prodrugs that require hepatic or intracellular metabolism for conversion to the active agent. As such, genetic variations in the involved enzymes, usually of the cytochrome P450 (CYP) family, can cause hypometabolism of these agents and diminish their efficacy^[41-43]^. These mutations or single nucleotide variations (SNVs) can be found at the germline or tumor somatic level. A different set of mutations and SNVs have been well described to be associated with drug hypermetabolism^[41,44-46]^. In particularly, variations in members of the CYP2 and CYP3A families have been associated with increased drug metabolism, decreased exposure of the tumor to the active agent, and decreased survival. Other involved proteins include glutathione s-transferases, thiopurine methyltransferase (TPMT), methylenetetrahydrofolate reductase, and UDP glucuronosyltransferase 1 family, polypeptide A1. These variations have been identified preclinically and clinically in patients with osteosarcoma^[47,48]^, medulloblastoma^[49]^, neuroblastoma^[50]^, acute lymphoblastic leukemia^[51-55]^. Many of these studies have been observation and association studies, however, requiring additional work to define the mechanisms of resistance (e.g., does hepatic or tumoral metabolism matter more?) and how to attack these mechanisms without increasing drug toxicity.

One approach taken in pediatric acute lymphoblastic leukemia (ALL) relates to TPMT. Allelic variation and enzymatic activity have been strongly correlated with response to 6-mercaptopurine and 6-thioguanine, with either excessive myelotoxicity (with hypoactive variants) or hypermetabolism and lack of efficacy at standard dosing (with hyperactive variants)^[56]^. As a result, TPMT testing has now become the standard of care for children with ALL at the start of therapy^[57]^. While additional pharmacogenomics biomarkers have been studied in pediatric cancers specifically^[58]^, the cost-benefit of testing of these genes continues to be evaluated. Testing of TPMT and other metabolism genes has focused primarily on dose reduction to avoid toxicity, but additional work needs to be done if pharmacogenomics testing could indicate safe dose escalation to overcome disease resistance.

### Drug efflux and influx

Drug efflux pumps function in both prokaryotes and eukaryotes, thought to act to clear endogenous or exogenous toxins from normal cells. Of these pumps, the ATP binding cassette (ABC) proteins function “actively”, i.e., by direct energy conversion and conformational change to expel bound molecules out of the cell. The prototypical ABC protein, MDR1, also known as P-glycoprotein or ABCB1, was identified as overexpressed in pediatric solid tumors and leukemias since 1986, with correlation with drug resistance and poor survival^[59-67]^. Other ABC proteins, including MRP1 and the ABCG proteins, have also been implicated in drug resistance in pediatric cancers^[68-79]^. Overexpression of these proteins in pediatric tumors is usually induced through other oncogenic pathways, such as MYCN amplification or overexpression in neuroblastoma^[60,80,81]^, CD133 expression in astrocytomas^[82]^, or SORCIN or MDK expression in acute lymphoblastic leukemia^[83,84]^. There has also been concern that the tumor cells upregulate ABC protein expression in response to therapy, resulting in higher expression during therapy or at recurrence^[60,85-87]^. Germline or somatic polymorphisms of these proteins have also been associated with increased activity, decreased drug exposure, and worse survival in a number of pediatric cancers^[74,88-91]^; polymorphisms have also been identified as a risk factor for cancer diagnosis^[90,92-94]^, suggesting there may be other functions of these proteins, as we discuss below. Prior studies generally evaluated expression and polymorphisms of the ABC genes proteins in low- or medium-throughput, however, and there is no clear pattern as to which of the 48 proteins may be expressed in a given tumor. As such, a comprehensive evaluation of these efflux pumps across tumor types is deserved.

In contrast to the large number of studies on drug efflux pumps, there has been very little research on drug influx and alterations that effect outcomes in pediatric cancer. Some work has been studied in the setting of adult cancers, such as platinum resistance due to mutations or downregulation of the *CTR1* gene^[95]^ or in regard to drug development generally^[96]^. As such, investigations into the drug influx mechanisms may be of value for pediatric cancer research.

P-glycoprotein itself has been evaluated as a therapeutic target in cancers generally, but with a single pediatric-specific Phase 1 study of tariquidar^[97]^, a specific inhibitor of this target. Tested in pediatric patients with refractory solid tumors, tariquidar decreased clearance of the taxane docetaxel and the vinca alkaloid vinorelbine, with some disease responses observed. Despite this promising result, it is unclear what the development plan is for this agent in children with cancers.

### DNA damage pathways

A number of chemotherapy agents act through DNA damage, which then relies on activation of the kinases ATR or ATM to activate p53, CHEK1, CHEK2, and other DNA damage response pathways. These pathways can then lead to either DNA repair or, more commonly, cell cycle arrest or apoptosis. These pathways are dependent on a number of additional interacting proteins, such as MDM2, which suppresses p53 activation, and multiple downstream proteins such as BRCA1, CDC25A, Rb, p21, CASP2, and CASP3, which are needed to allow either DNA repair, cycle arrest or apoptosis (reviewed in^[98]^). Thus, there are multiple tumor suppressors which can be targeted in cancer cells to avoid DNA-damage-induced cytotoxicity.

*TP53*, which encodes the p53 protein, is somatically mutated in < 5% of all pediatric cancers at diagnosis^[13]^, with a notable exceptions in osteosarcoma^[99]^ and adrenocortical carcinomas^[100]^. Germline mutations, such as in Li Fraumeni Syndrome, are associated with increased cancer risk and increased toxicity from DNA damaging agents^[101]^. However, somatic *TP53* mutations or epigenetic silencing have been associated with increased rates of relapse, with enrichment in these tumors, and with worse prognosis both at diagnosis and relapse, including in glioma^[102,103]^, neuroblastoma^[104-107]^, and leukemia^[108,109]^. Mutations in other components of the DNA repair and cell cycle pathways in refractory pediatric cancers, including ATM, ATR, PTEN, and CHEK1, have also been associated with poor prognosis, presumably through avoidance of cell death^[110-112]^.

Indirect mechanisms are more commonly found to repress the DNA damage pathways, promoting cell survival and therapeutic resistance. MYCN and MYC basally can upregulate *TP53* expression, but when overexpressed, either by amplification, translocation, or epigenetically-driven overexpression, these transcription factors can promote MDM2 expression, which ubiquitinates p53 leading to its degradation. MDM2 is overexpressed in pediatric tumors including leukemias^[113-115]^, neuroblastoma^[116]^, retinoblastoma^[117]^ and associated with poor prognosis and/or treatment resistance^[118-121]^, supporting its role in chemoresistant disease. Additional response pathways, such as MEK/ERK activation and NF-kappaB activation, are being studied in pediatric cancers.

There is also a paradoxical role of DNA repair proteins in chemoresistance. DNA damage can activate p53 to induce apoptosis, if the damage is sufficient, or cell cycle arrest and DNA repair, which would maintain viability. Expression of proteins including CHEK1, CHEK2, ERCC1, ERCC2, PARP and WEE1 predispose cancer cells treated with chemotherapy toward DNA repair and proliferation. As such, these proteins have been identified as either prognostic biomarkers or therapeutic targets to improve chemotherapeutic effects in pediatric tumors^[122-132]^. The mechanisms that balance the expression and stability of these proteins continue to be areas of active investigation.

As the roles of DNA repair pathways in pediatric chemoresistance have been elucidated, opportunities to use targeted therapies have arisen. Many of these drugs were developed originally for cancers in adults, but there are ongoing pediatric early phase studies of PARP inhibitors^[133]^ and WEE1 inhibitors^[134]^, alone or in combination with chemotherapy.

### Apoptosis via cytochrome C, redox/ROS activation, and caspase activation

Apoptosis can be triggered by a number of different pathways in normal cells. DNA damage can activate p53 to promote expression of pro-apoptotic members of the BCL2 family, NOXA and PUMA. These proteins then translocate to the mitochondria and bind antiapoptotic BCL2 proteins, including BCL2, BCL2L1, and MCL1. This process releases the proteins BID, BIM, BAK and BAX to then release cytochrome C and apoptosis inducing factor (AIF) into the cytosol, which then activates caspase cleavage and apoptosis. Alternatively, the production of reactive oxygen species can cause direct membrane damage of organelles, directly releasing AIF and cytochrome C into the cytosol. New signaling pathways involving NFE2L2^[135-137]^ (aka NRF2) and AKR1C^[138]^ have also been implicated in modulation of and resistance to ROS/redox-mediated cell death. Additionally, the “extrinsic” pathway can be triggered to induce apoptosis; external ligands, such TRAIL, TNFA, or FASL can bind their respective receptors, which then can directly activate CASP8 to trigger apoptosis.

Altered BCL2 family expression occurs in a number of childhood cancers. Overexpression of BCL2, BCL2L1, and MCL2 can stoichiometrically inhibit NOXA and PUMA, effectively squelching apoptosis. This overexpression can be driven by chromosomal translocations, through signal transduction from extracellular signaling^[139]^, alternative splicing^[140]^ or epigenetic dysregulation^[141]^. Antiapoptotic BCL2 family expression has been seen in numerous pediatric cancers, including neuroblastomas^[142-144]^, leukemias^[141,145-147]^, lymphomas^[148,149]^, brain tumors^[150-152]^, and sarcomas^[153-156]^.

Investigations into caspase expression and processing, in contrast, have variably linked these pathways to therapeutic resistance and poor survival, particularly in leukemias and solid tumors^[157-163]^. However, the numerous mechanisms that can activate caspase processing and apoptosis have riddled these studies with confounders, suggesting that effects on caspase biology are likely secondary to other oncogenic pathways. Additionally, there are few opportunities to target this pathway therapeutically. As a result, this area of study has largely fallen by the wayside.

From a therapeutic perspective, BCL2 and MCL1 serve as potential targets, alone or in combination with chemotherapy. Venetoclax (ABT-199) and navitoclax (ABT-263) inhibit each protein respectively, and are currently in early phase pediatric clinical trials. Venetoclax specifically has received FDA approval in adult AML and CLL, alone or in combination with one additional drug^[164-166]^. However, in the pediatric approach, monotherapy is not expected to be effective alone; as such, a novel Phase 1 study has been opened that rapidly allows evaluation of venetoclax in monotherapy and combination chemotherapy^[167]^ This novel approach advocates for the advancement of a targeted therapeutic approach for pediatric cancer patients specifically.

### Epigenetic and miRNA-mediated resistance in pediatric cancers

As noted above, pediatric cancers differ from adult cancers in their comparatively quiet genomes; instead, epigenetic dysregulation is of greater impact on disease biology^[14,168,169]^. Broadly, this includes effects on DNA methylation, histone methylation, and histone acetylation, with differential effects depending on the pathway targeted and proteins involved. Implicated proteins, either due to direct mutation, overexpression, or due to novel interactions with other oncogenes, include KMT2A^[170,171]^ (aka MLL), DOT1L^[172]^, EZH2^[173-175]^, KDM1A^[176-178]^ (aka LSD1), CREBBP^[179,180]^, SWI/SNF, SETD2^[181,182]^, ATRX^[183,184]^, and H3F3A^[185-187]^ (aka Histone 3.3) mutations, to name a few, as well as the HDAC family^[188-192]^. Most of these proteins have effects across the genome, so elucidation of specific effects is complex. Nonetheless, they have been viewed as key factors that maintain tumor viability in the face of conventional chemotherapies. As such, novel agents targeting these epigenetic modifiers have been developed specifically for pediatric cancers.

In MLL-rearranged ALL, DOT1L was found to be a key driver of disease aggression; the DOT1L inhibitor pinometostat was studied in a pediatric Phase 1 trial, with no objective responses seen in single agent use^[193]^. There are a number of studies of the HDAC inhibitor vorinostat, alone or in combination, in pediatric cancer patients, though with generally low rates of responses in single use^[194-198]^. Tazemetostat is an EZH2 inhibitor currently under study in pediatric patients with lymphomas, synovial cell sarcoma, or other relapsed/refractory solid tumors^[199]^. The most novel agent currently in a pediatric Phase 1 study is the LSD1 inhibitor, seclidemstat (SP-2509), which was specifically identified for its efficacy against Ewing sarcoma^[200]^. It is likely that these agents will need to be used in combination therapies, as a common challenge for antiepigenetic therapies has been their slow effect, but they still offer novel approaches for the treatment of pediatric cancers.

In parallel to the study of epigenetics of pediatric cancers, the roles of miRNAs are also being evaluated. Specifically, miRNAs have been evaluated as mediators of therapeutic resistance in childhood leukemias^[201]^, neuroblastoma^[202]^, and CNS tumors^[203]^. However, in contrast to epigenetic modifiers, therapeutic options to inhibit or sponge miRNAs remain in early development, given the challenges of drug targeting and delivery, and no studies yet exist in pediatric oncology. Still, this remains a potential area for study.

### New therapies, new mechanisms of resistance

Targeted antineoplastic therapies have been over 20 years, generally falling into two large categories: (1) small molecules targeting specific cellular proteins with inhibitory effects; and (2) monoclonal antibodies targeting cell-surface proteins to inhibit their functions and/or to recruit an anti-tumor immune response. Since their first use in cancers, resistance mechanisms have been identified, including in pediatric cancers. One of the first targeted therapies brought to clinic were inhibitors of MTOR, a key component of the PI3K/AKT oncogenic pathway. These agents, including sirolimus, everolimus, and temsirolimus, were found to be comparatively less toxic than conventional chemotherapies and with some benefits, generally in combination use, against a variety of cancers^[204-208]^. However, these responses were generally found to be not durable. In some tumors, the MTOR proteins were found to develop a gain-of-function mutation that rendered them immune to inhibition by these first and second-generation inhibitors^[209]^, though third-generation inhibitors are now being developed to sidestep these mutations. More commonly, however, cancers were found to overcome MTOR inhibition by activation of other oncogenic pathways, including overexpression of MYC^[210]^, IDO1^[211]^, other components of the AKT pathway^[212]^, or rebound activation of AKT^[213,214]^, such as through MTORC2 activity (not inhibited by current MTOR inhibitors)^[215-217]^.

A rapidly expanding class of drugs with increasing use in pediatric oncology are kinase inhibitors, including receptor tyrosine kinase inhibitors and other kinase inhibitors targeting intracellular pathways. The development of imatinib, an inhibitor of the BCR-ABL fusion tyrosine kinase, revolutionized the treatment of chronic myelogenous leukemia^[218]^ and other leukemias expressing this fusion protein^[219]^. While these kinase inhibitors are generally designed for specificity for one kinase, often times they have effects on multiple kinases due to homology among the proteins, with varying toxicity and efficacy^[220-223]^. With these drugs, resistance often develops with time as well. A common mechanism of resistance is the isolation of a new subpopulation with a mutation resistant to the original drug, such as identified in BCR-ABL in leukemias^[224-226]^ and ALK in neuroblastoma^[227,228]^ (the development of these mutations are described below). In these cases, multiple second and third generation inhibitors have been developed against the same target, allowing for cycling among drugs for durable remission. In other cases, the cause of resistance has been less clear because of the new use of these agents, but extrapolation from adult data suggests resistance could be due to activation of an alternative oncogenic pathway^[229-231]^, increased drug efflux^[232]^, or downregulation of apoptosis^[233,234]^. As such, sequential or concurrent multiagent therapy may be needed for durable efficacy.

Monoclonal antibodies, included engineered antibodies with or without conjugated drugs, have been developed against a variety of cell surface targets in pediatric cancers^[235-241]^, generally with success but with some notable failures^[242-246]^. In the latter cases, activation of alternative pathways generally was responsible for lack of efficacy. However, two other important mechanisms of resistance have been identified. In some cases, the cancer became resistant to the monoclonal antibody by losing expression of the target antigen, such as CD20 or CD30 in lymphomas^[247,248]^. This is incidentally a mechanism of resistance for the first-in-class anti-CD19 chimeric antigen receptor (CAR) T-cells^[249]^. In other cases, neutralizing antibodies have developed against the therapeutic antibody, with diminished pharmacokinetics and response^[250-252]^. These neutralizing antibodies are also being identified against other therapeutics, such as asparaginase used against acute lymphoblastic leukemia^[253-255]^, though impact on drug efficacy is variable. Different approaches are being developed against these resistance mechanisms, including combination agent therapies to prevent activation of alternative oncogenic pathways, antibodies capable of multi-antigen binding, and recombinant antibodies and drugs with diminished anti-antibody production.

### Future areas of study in pediatric cancer treatment resistance, and key collaborations for advancement

New mechanisms of therapeutic resistance continued to be identified as our understanding of cancer biology deepens. A major question has been the origin of the chemoresistant population, particularly regarding the existence of cells with resistance mutations as a subpopulation from diagnosis or the generation of novel mutations over time. In neuroblastoma, there is evidence to support both mechanisms. Next-generation deep sequencing approaches have allowed for higher resolution identification of tumor subpopulations; sequencing on paired diagnosis-relapse patient samples has demonstrated that mutations identified in relapse samples can also be identified at diagnosis but below the resolution of traditional testing^[256,257]^. In contrast, a study examining ALK mutations in cell lines derived from a patient with neuroblastoma at original diagnosis and at relapse. In these paired cell lines, different ALK mutations were identified, with differential response to ALK inhibitors^[258]^. However, it is unclear if these mutations may have occurred ex vivo. These different mechanisms have major implications on diagnostic and therapeutic approach; the presence of cancer subpopulations would support deeper sequencing of tumors and/or empiric use of ALK inhibitors to squelch selection of a resistant population, whereas the generation of de novo mutations would argue against these efforts.

An analogous though more contentious topic has been the role of “cancer stem cells” in therapeutic resistance. These stem cells have been variably defined phenotypically but include the capacity to initiate a new tumor, a slower proliferation rate and/or maintenance of a basal undifferentiated population. These features can inherently induce therapy resistance, as most chemotherapeutic agents rely on damage to the mechanisms of cell proliferation. There is some question about the relevance of “cancer stem cells” in different tumors, but there is also some evidence that markers of these cells can promote therapeutic resistance. For example, CD133, a surface marker associated with stem cells in a number of pediatric solid tumors and leukemias^[259-261]^, can promote therapeutic resistance directly and has been associated with poor survival clinically^[82,262-265]^. Additional work is needed to clarify the causality of these stem cell features in disease aggression.

Intercellular effects within the tumors also have demonstrated impacts on disease resistance. The tumor microenvironment is a complex landscape of primary neoplastic cells and recruited supporting cells, including immune populations, fibroblasts, and vasculature. Cell signaling among these cells is complex and includes interactions through cytokines^[266-269]^, extracellular vesicles (reviewed in^[270]^), and even the efflux of chemical signaling via ABC transporters^[271,272]^. The role of tumor immunosuppressive cells, such as tumor-associated macrophages^[273-275]^, regulatory T cells^[276-279]^, myeloid-derived suppressor cells^[280-282]^, continues to be evaluated in pediatric cancers, and these cells undoubtedly have roles in therapy resistance beyond immunotherapies.

Advances in pediatric cancers have been generally possible only through collaborations between scientists and clinicians around the world, including advances against treatment resistance. The Pediatric Preclinical Testing Program has been an important contributor to this work, with the first comprehensive evaluations of existing and novel therapeutics for childhood cancers^[283]^. Now the Pediatric Preclinical Testing Consortium, http://www.ncipptc.org/, this group builds on the basic science discoveries of pediatric oncology to elucidate the mechanisms of disease biology in childhood cancers, the efficacy of novel drugs and drug combinations, and the potential roles of these agents in clinical trials and use. This international work is but one of many groups around the world, including the Children’s Oncology Group, The International Society of Pediatric Oncology, and numerous childhood cancer research groups and early phase consortia. Collaboration among these private groups, federal agencies, and pharmaceutical companies will further optimize existing treatments and add in novel therapeutics to overcome treatment resistance in pediatric cancers.

## Conclusion

As advances continue to be made in therapeutic approaches for pediatric cancers, known and new mechanisms of therapeutic resistance will need to be considered. Known intracellular mechanisms of resistance can be targeted with new adjunctive therapies to ensure proper drug delivery and retention and optimal induction of apoptosis. New therapeutic approaches will also need to consider the genetic and epigenetic changes that can be selected and/or induced in response to therapy, and adjunctive approaches attacking newly discovered mechanisms of resistance, including effects on the tumor microenvironment, must be designed to further improve therapy. These multidimensional therapeutic approaches will offer the next great leap forward in outcomes for children with pediatric cancers.

## Declarations

### Copyright

© The Author(s) 2019.
